# Damage control Surgery – physiopathological benchmarks - Part I

**Published:** 2008-04-15

**Authors:** Beuran Mircea, Iordache Florin-Mihail

**Affiliations:** * Department of Surgery, Emergency Clinical HospitaL

**Keywords:** damage control surgery, trauma, hypothermia, hypocoagulability, acidosis

## Abstract

The following article, submitted in two complementary parts deals with an important and also modern concept developed under the name of damage-control surgery. Physiopathologically, the multiple injured patient is characterised by the probable, not just possible, appearance of the „blood’s vicious cycle” of hypocoagulability, hypothermia and acidosis with death as a result. The first part of the article addresses the changes that are the reasons and the basis for applying damage-control surgery. Hypothermia is a direct result of trauma and patient’s exposure to it but can also emerge throughout transportation, evaluation, emergency and surgical procedures to which the patient undergoes. Surgical procedures are directly a source that decreases the core temperature. While blood losses accompany trauma for certain and affect clot formation, the patient’s coagulation system is impaired by these losses and the dysfunction is further enhanced by hypotermia, different mechanisms being involved. The third lethal component is acidosis. While being at first metabolically produced because of tissular injury, it is further enhanced by the other two elements. From a practical point of view, hypothermia and hypocoagulability can be though, more teoretically addressed, acidosis is more difficult to correct. As fav as the emergency specialist is concerned for the moment, the best solution to deal with this deadly triad is to prevent it. Damage-control surgery is just one type of measure in the process of prevention.

Trauma represents an issue with global impact. In 2000, trauma was the cause of approximately 5 mil deaths, trauma having a death rate of 83 per 100,000 people, also representing 9% of the global death rate.

In the 90s, new definitions of certain concepts regarding the specific physiopathology of trauma patients hae lead to new methods of therapeutic approach. One of the modern approaches is damage control surgery.

The trauma patient usually has an active haemorrhage, often of multiple origins. If he is subjected to corrective surgery, a timely intervention and blood loss can lead to the so called “lethal triad” (hypocoagulation, hypothermia and acidosis). This combination has also been called the “bloody vicious cycle” by Moore et al. Once the lethal triad is in place, death rate exceeds 90%. The elements of the lethal triad sustain each other in a vicious cycle which increases the already existent problems.

In addition to these 3 elements, Asensio et al has also named dysrhythmia as a “true herald of patient death”. Therefore, we can talk about a “lethal tetrad” in traumas. All 3 elements of the lethal triad have synergic effects: initial hypothermia is emphasized by blood loss which, in turn, accentuates haemorrhage if it’s not kept under control.

We will now analyse the elements of the lethal triad.

**Figure 1:**
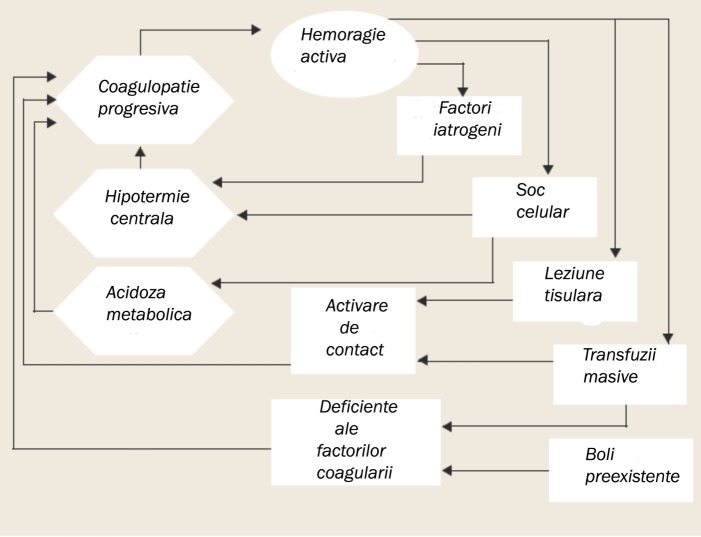
The vicious circle of acidosis, coagulopathy and hypothermia - After Moore EE, Staged laparotomy for the hypothermia, acidosis, and coagulopathy syndrome - *Am J Surg* 1996, 172, 405-410

## Hypothermia

Hypothermia is a very high risk factor for trauma patients and is defined by a decrease in central temperature under 36ºC. The effects of hypothermia in traumas are often underestimated. Its causes are detailed below in **Table 1**:

**Table 1:** Causes of hypothermia in trauma patients (after E, Smith CE)

Causes of hypothermia in trauma patients
1. Disturbance of thermo regulating mechanisms:
a. Trauma
b. Hypovolemic shock
c. SNC lesions
d. Extreme age points
e. General or neuroaxial anaesthetic
f. Related illnesses (diabetes, cardiac failure)
g. Alcohol or antidepressant use
2. Extreme heat loss
a. Prolonged exposure to the environment
b. Blood perfusions and intravenous drips
c. Burns
d. General or neuroaxial anaesthetic

A recent study undertaken on 288 severe trauma patients revealed that the body temperature of nearly half of them (49.6%) had different degrees of hypothermia. The same study revealed that the risk of developing hypothermia increased for patients over 65 years old and for those cases where patients were stuck at the scene of the accident. The authors also noted a reduced frisson effect on only 4% of these patients. This confirms that trauma patients have a more reduced capacity of adjusting to heat loss. Another study, by Jurkovich et al, shows the direct correlation between hypothermia and trauma patient death. This study showed that a high severity of lesions, the need for a high quantity of intravenous drips as well as shock all contributed to increased hypothermia. Other studies had similar results.

Hypothermia can take place not only in the early stages of a trauma patient, it can appear along the course of the condition - from the scene of the accident, to the emergency department or even the operating theatre. Over 90% of patients develop hypothermia in the initial stages of therapy, usually whilst in the emergency department. Hypothermia can appear or increase within the operating theatre as well, due to the interference of anesthetics with the patient’s own thermo-regulating mechanisms. In addition, surgery leads to important heat loss due to cold rooms and cold intravenous drips administered during therapy. A prolonged surgical intervention represents therefore an augmenting factor of hypothermia.

Hypothermia in turn, increases hypocoagulability. This is produced through the following mechanisms: inhibition of the blood platelets function, and direct interference with the flow of coagulation. The enzymes involved in clot forming, which can be either platelet’s or plasma’s, are dependant on normal temperatures for a correct activation. Hypothermia also leads to exaggerated fibrinolysis. 

Hypothermia also has a depressing effect on the myocardium and on its contracting functions, especially on the performance of the left ventricle and also on the cardiac rhythm.

## Hypocoagulation

The causes that lead to hypocoagulability are varied and interrelated (**Table 2**):

**Table 2:** Causes of hypocoagulation in traumas

• Consumption and dilution of coagulation factors
• Consumption and dilution of platelets
• Disturbance of the coagulation flow by intravenous drips, blood derivatives or hypocalcemia.
• Fibrinolysis activation
• Disseminated intravascular coagulation

Volume replacement solutions, usually crystalloids, but not only, also determine a sanguine dilution, this way interfering with coagulation. A great part of these solutions interfere directly with coagulation mechanisms as well. On the other hand, sanguine derivative drips never re-establish coagulability - they can even accentuate it. Moreover, the criteria for administering sanguine solutions (blood, erythrocytes or thrombocytes, etc) are not completely standardized yet. There are different views regarding their practical use and their association system, especially when aiming to re-establish coagulation. A very important issue underlined by a recent study shows that hypocoagulability appears in the early stages of trauma cases and is not only a result of replacement solutions. Hypocoagulability in turn, directly increases haemorrhage.

In order to prevent hypocoagulability, the source of the haemorrhage needs to be kept under control. This is done mainly surgically or through other methods (angioembolisation)

## Acidosis

Acidosis is defined as a decrease of the sanguine pH level of under 7.36. In trauma, acidosis is often metabolic. In the early stages, acidosis is caused by tissue damage and shock. In turn, acidosis amplifies hypocoagulability but also interferes with other systems and mechanisms. Acidosis is very serious for trauma patients. It can be evaluated by using different parameters. The most commonly used one is lactic acid. Lactic acidosis is very high in trauma cases and can be used as an indirect lesion and shock indicator. However, not all authors agree, some considering base deficit as a more useful practical indicator. Acidosis, an element of the lethal triad, appears quite late in the stages of trauma cases and is more difficult to rectify.

To simplify, the results of these trauma cases realities can be summarized in the following table of valid truths (**Table 3**):

**Table 3:** Some truths in trauma (after T. Scalea **15**)

• Only haemorrhage can kill instantly, intestinal lesion contamination can be rectified at a later stage
• In trauma cases, everything lasts longer than originally believed
• Due to the pace given by the circumstances, some lesions may not be done in laparotomy
• Hypothermia, acidosis and coagulopathy lead to the same outcome
• Polytrauma patients need to be in intensive care

All of the above have lead to the new, or more likely, the up-to-date concept of damage control therapy, based on physiopathological rectification of issues. 
